# Factors determining family planning in Catalonia. Sources of inequity

**DOI:** 10.1186/1475-9276-11-35

**Published:** 2012-07-20

**Authors:** Carme Saurina, Laura Vall-llosera, Marc Saez

**Affiliations:** 1 Research Group on Statistics, Econometrics and Health (GRECS), University of Girona, Campus de Montilivi, 17071, Girona, Spain; 2 CIBER of Epidemiology and Public Health, Campus de Montilivi, 17071, Girona, Spain

**Keywords:** Inequalities, Family planning, Immigration, Mixed models

## Abstract

**Introduction:**

In recent decades, the foreign population in Spain has increased significantly, particularly for Catalonia, an autonomous region of Spain (2.90% in 2000 and 15.95% in 2010) and in particular Girona province (6.18% in 2000 and 21.55% in 2010). Several studies have shown a lower use of family planning methods by immigrants. This same trend is observed in Spain. The objective of this paper is to determine the existence of differences and possible sources of inequity in the use of family planning methods among health service users in Catalonia (Spain) by sex, health status, place of birth and socioeconomic conditions.

**Methods:**

Data were taken from an ad-hoc questionnaire which was compiled following a qualitative stage of individual interviews. Said questionnaire was administered to 1094 Catalan public health service users during 2007. A complete descriptive analysis was carried out for variables related to public health service users’ sociodemographic characteristics and variables indicating knowledge and use of family planning methods, and bivariate relationships were analysed by means of chi-square contrasts. Considering the use (or non-use) of family planning methods as a dependent variable and a set of demographic, socioeconomic and health status variables as explanatory factors, the relationship was modelled using mixed models.

**Results:**

The analysed sample is comprised of 54.3% women and 45.7% men, with 74.3% natives (or from the EU) and 25.7% economic immigrants. 54.8% use some method of family planning, the condom (46.7%) and the pill (28.0%) being the two most frequently used methods. Statistical modelling indicates that those factors which most influence the use of family planning methods are level of education (30.59% and 39.29% more likelihood) and having children over 14 (35.35% more likelihood). With regard to the origin of the user, we observe that patients from North Africa,sub. Saharan Africa and Asia are less likely to use family planning methods (36.68%, 38.59% and 70.51%, respectively).

**Conclusions:**

The use of family planning methods is positively related to a higher level of education and having children over 14. Factors such as sex, age, income and self-perceived health do not appear to influence their use. Furthermore, being a native of this country, the European Union or Central/South America represents a greater likelihood of use than being African or Asian. Although no general differences in use were found between sexes, the difference found in the case of Asian women stands out, with a higher likelihood of use.

## Introduction

In recent decades, the foreign population in Spain has increased from 1.60% of the total population in 1998 to 12.20% in 2011 [1]. Changes in the annual rates are even greater for Catalonia, an autonomous region of Spain, (2.90% in 2000 and 15.95% in 2010) and in particular Girona province (6.18% in 2000 and 21.55% in 2010) [2].

Inequity in access to and use of different contraceptive methods affects human rights [3]. Although for immigrants from low-income countries or countries with deficient health services migrating may provide a greater opportunity to access sexual and reproductive health services in the host country, it is often the different barriers to access (such as difficulty with the language, dealing with health professionals or lack of information) in addition to structural, social and cultural factors, that determine real opportunities to use said services.

Numerous studies have analysed the situation regarding the use of family planning methods in developing countries [4-13] and developed countries [14-18], and studies also exist on differences between native and immigrant populations of developed countries. However, talking about inequities in the use of family planning methods is more difficult than talking about health inequities in general [19-21], because, in addition to objective factors regarding knowledge of and access to said methods, there is also the influence of moral issues, such as the fear of transgressing certain taboos, cultural factors and even religious impediments. In developing countries, and particularly African countries, it is important to take into account that the attitudes towards and use of contraception may vary due to a multitude of factors, including the quality of the health service and its professionals, availability of and access to family planning methods [22], level of wealth [23], gender roles and the socioeconomic context of the country [20] . Discrepancies have also been found in developing countries with regard to interest in modern family planning methods and their real use. Some results suggest that more support is required to ensure greater prevalence of use, in terms of access to different methods and from an educational and cultural viewpoint [16], as well as greater involvement by health professionals [17].

Research into differences in the use of family planning methods between immigrant and native populations in different countries generally indicates less use by immigrants [24-26], even when they have resided in the host country for some time [21] . This same trend is observed in Spain, with less frequent use of the health service by the immigrant population when it comes to the general health service [27-29] and family planning clinics and the use of different methods of contraception [30,31], especially for certain groups of immigrants.

The main aim of this study is to detect sources of inequity by analysing factors influencing the use or non-use of family planning methods in a geographical context where there is a high level of relatively recent immigration and a public health system which offers universal cover. The consequences of less access to family planning services are not only a higher number of undesired pregnancies but also increased risk of infections and sexually transmitted diseases, and higher numbers of abortions [14,15,25].

## Methods

The data used in this study were taken from a questionnaire administered to a sample of Catalan health service users. The health care system in Catalonia has all the powers derived from the Spanish constitution. The Spanish model decentralizes the responsibility for health to autonomous communities.

It is a validated ad-hoc questionnaire with copyright n 02/2010/2833 [32]. The questionnaire was compiled following a qualitative stage involving the conducting of 37 individual interviews to users, health professionals and cultural mediators aimed at drawing out sensitive information and determining opinions regarding the possible existence of access barriers to a public and universal health service such as the one in Spain. The questionnaire was administered to 1094 public health service users at health centres in Girona (ABS Salt and Santa Caterina Hospital), BaixEmpordà (Palamòs Hospital and Palafrugell ABS), Barcelona (Hospital del Mar, Raval Sur ABS, Vila Olímpica ABS and Besòs ABS) and Lleida (Arnau de Vilanova Hospital, RamblaFerran ABS and Eixample ABS) in 2007. Point transect sampling was used in order to ensure the representativeness of the sample [29] . Given the aim of the article, only users aged between 15 and 49 were selected, which meant working with a total of 876 complete questionnaires. Data were weighted in order to correctly reflect the distribution of the population of users according to origin.

Data analysis comprised a complete descriptive analysis of variables relating to the sociodemographic and health status characteristics of public health service users and variables indicating their knowledge and use of family planning methods (Tables 1, 2 and 3). Bivariate relationships were analysed by means of chi-square contrasts between the variable indicating use or non-use of family planning methods and the variable addressing the type of method used and users’ sociodemographic variables and health characteristics (Tables 4 and 5). Finally, considering the use (or non-use) of family planning methods as a dependent variable and a set of demographic, socioeconomic and health status variables as explanatory factors, the relationship was modelled using mixed models.

**Table 1:** Descriptive demographic and socioeconomic variables

**Characteristics**	Number of cases (n)*	Percentage (%)**
**Sex**		
Male	414	45.7
Female	462	54.3
**Place of birth**		
Native	346	69.1
European Union	53	5.2
Eastern Europe	40	3.4
Central and South America	163	7.2
North Africa	136	6.8
Sub-Saharan Africa	67	2.2
Asia	71	5.9
**Level of education**		
Primary school unfinished	229	24.3
Secondary school finished	424	50.5
Higher	221	25.2
**Having children under 14**		
No	131	26.5
Yes	301	73.5
Missing data	444	55.9
**Suffering chronic illness**		
No	647	72.6
Yes	224	27.4
**Self-perceived health status**		
Poor	22	2.1
Normal	180	18.2
Good	367	44.3
Very good	235	28.3
Excellent	67	7.1

*Unweighted n’s and ** weighted percentages (%).

**Table 2:** Distribution of users according to age group

**Distribution by age in quartiles**	Number of cases (n)*
Aged 15-25	202
Aged 26-31	204
Aged 32-38	197
Aged 39-49	207

*Unweighted n’s (%).

**Table 3:** Type of planning methods used among those who use them

**Use of family planning methods**	**Permanent methods**	**IUD/vaginal ring**	**Pill**	**Condom**	**Other: Traditional, injections, patches, implants, morning after pill,…**
Number of cases (n)*	43	45	136	203	29
Percentage (%)**	10.2	9.4	28.9	46.7	4.9

Missing data (n=420, 45.2%). *Unweighted n’s and ** weighted percentages (%).

**Table 4:** Relationship between the use of family planning methods and user characteristics

**Use of family planning methods**	**Chi-square (V Cramer)**	**p-value**	**Interpretation (use less)**
Age in quartiles	10.959 (0.117)	0.012	Aged 39-49
Place of birth	37.361 (0.216)	0.000	European Union and Central-South America use more Africans and Asians use less
Level of education	8.630 (0.104)	0.013	Primary education lessHigher education more
Basic origin	4.915 (0.078)	0.000	Immigrants
Having children under 14	10.971 (0.176)	0.001	Not having children

No differences with regard to sex, type of family planning methods used, chronic illness, self-perceived health and income.

**Table 5:** Relationship between the type of family planning methods used and user characteristics

**Type of family planning methods used**	**Chisquare (V Cramer)**	**p-value**	**Interpretation**
**Sex**	43.377 (0.313)	0.000	Men: more condoms
			Women: more pill and other
**Age in quartiles**	84.196 (0.252)	0.000	Aged 15–38: less permanent methods
			Aged 39–49: more permanent methods and IUD
			Aged >45: more permanent methods
**Level of education**	19.248 (0.147)	0.014	Primary: more permanent methods and other
			Secondary: more condoms less others
			Higher: less permanent methods
**Place of birth**	37.285 (0.145)	0.041	Natives: more permanent methods, condoms
			European U: more condoms
			less IUD and permanent methods
			Eastern Europe: less IUD and other
			Central-South America: more IUD-ring and other
			North Africa: more pill
			Sub-Saharan Africa more other
			Asia: more other
**Children under 14**	9.467 (0.226)	0.050	Yes: more IUD/vaginal ring
			less permanent methods
**Self-perceived health**	29.451 (0.129)	0.021	Better: more IUD
			Worse: more permanent methods
**Chronic illness**	9.434 (0.146)	0.051	Yes: more permanent methods
			less condoms

No differences with regard to basic origin and income.

Other: Traditional, injections, patches, implants, morning after pill,…

The model studies factors that have a greater or lesser influence on the likelihood of using family planning methods. Thus, the model to be estimated is specified as follows [eq.(1)]:

(1) $$\ln\left(\frac{\mathrm{Pr}\left(Y_{i}=1\right)}{1-\left[\mathrm{Pr}\left(Y_{i}=1\right)\right]}\right)=\beta_{0i}+\sum_{j=1}^{J}\beta_{j}X_{\mathrm{ji}}$$

where Y is the response variable, that is, use or non-use of family planning methods and Pr(Y=1) refers to the likelihood of using family planning methods and is modelled by a logistic regression. X refers to the group of users’ sociodemographic and health status variables. The special feature offered by this model is that it introduces the heterogeneity $\beta_{0i}=\beta_{0}+\eta_{1}$associated with the user through a normal random effect, where $\eta_{l}\to \left(0,\tau_{\eta }\right)$. This modification allows us to collect the individual effects that are not explained by the explanatory variables of the model (those that depend uniquely on the patient). Due to the multiple advantages it provides within the context of this study, the aforementioned models are estimated using the Bayesian approach [33-35]. All computations were carried out using the interface INLA, running directly in R (version R 2.11.0) [36].

Different effects were tested, both fixed and random, in order to determine the final specification of the model. Models were compared using the DIC (Deviance Information Criterion) and the conditional predictive ordinate (CPO) for each observation (in fact – mean (log(cpo)). CPO is a cross-validated predictive approach, i.e. predictive distributions conditioned on the observed data with a single data point deleted. Asymptotically the CPO statistic has a similar dimensional penalty to AIC. From this perspective, the CPO statistic may be similar to DIC. In both cases, the lower the DIC or the CPO, the better the model. The finally selected model is shown in Table 6.

**Table 6:** Model results

	**RR (95%CI)**^**1**^
AGE	
Aqeq1 (15–25)	1
Ageq2 (26–31)	0.9779 (0,7521 – 1.2485)
Ageq3 (32–38)	0.8544 (0.6477 – 1.1043)
Ageq4 (39–49)	0.7977 (0.5899 – 1.0502)
SEX	
male	1
female	0.8954 (0.6711- 1.1724)
ORIGIN	
Native	1
European Union	1.3619 (0.7237 – 2.226)
Eastern Europe	0.8820 (0.3334 – 1.7408)
Central and South America	1.1707 (0.7921 – 1.6478)
North Africa	0.6332 (0.3947 – 0.9369)
Sub-Saharan Africa	0.6141 (0.3342 – 0.9805)
Asia	0.2949 (0.1202 – 0.5580)
EDUCATION	
Primary	1
Secondary	1.3059 (1.0028 – 1.6807)
University	1.3929 (1.0302 – 1.8460)
Interaction sex*origin	
Male and native?	1
Female and European Union	0.9433392 (0.4328 – 1.8276)
Female and Eastern Europe	0.9045 (0.2802 – 2.2907)
Female and Central and South America	0.9740 (0.5902 – 1.5213)
Female and North Africa	1.1367 (0.5794 – 2.0090)
Female and sub-Saharan Africa	1.4626 (0.4911 – 3.2330)
Female and Asia	3.3542 (1.1195 – 8.0612)
Children over 14	
NO	1
YES	1.3535 (1.0917 – 1.6564)
Self-perceived health	
Poor	1
Normal	0.8468 (0.6303 – 1.1466)
Good	0.8808 (0.6835 – 1.1610)
Very good	0.8951 (0.6816)
Excellent	1.0960 (0.7763 – 1.5374)
Number of chronic illnesses	
None	1
One	1.1264 (0.8722 – 1.4206)
More than one	1.0289 (0.6353 – 1.5214)
Individual heterogeneity (sd)^2^	0.010427554
DIC	1491.48
Effective number of parameters	26.68
-log(mean(cpo))	0.8586142

^1^ Mean (95% Credible Interval) 2 Mean (Standard Deviation).

## Results

The main descriptive results shown in Tables 1,2 and 3 indicate a weighted percentage of 54.3% of women and 45.7% of men. 74.3% are natives or from EU countries, and 25.7% economic immigrants (according to the UNDP country classification [37]). 24.3% state having a low level of education, while 25.2% have studied in higher education. With regard to health status, 27.4% state they have a chronic illness while 20.3% perceive their health to be normal or poor. 54.8% of users in the sample state they regularly use some method of family planning, with the pill (28.9%) and the condom (46.7%) the most common.

**Figure 1:**
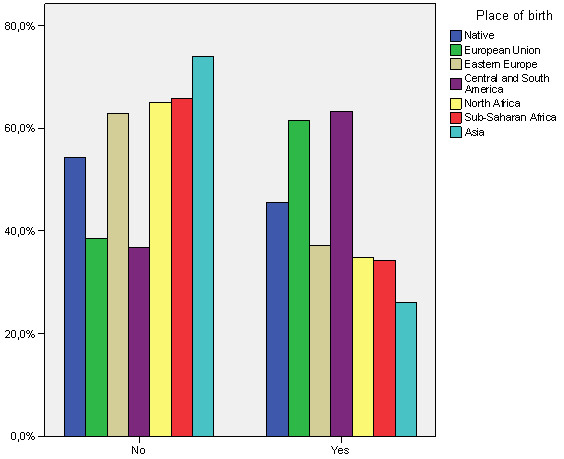
Relationship between the use of family planning methods and place of birth.

**Figure 2:**
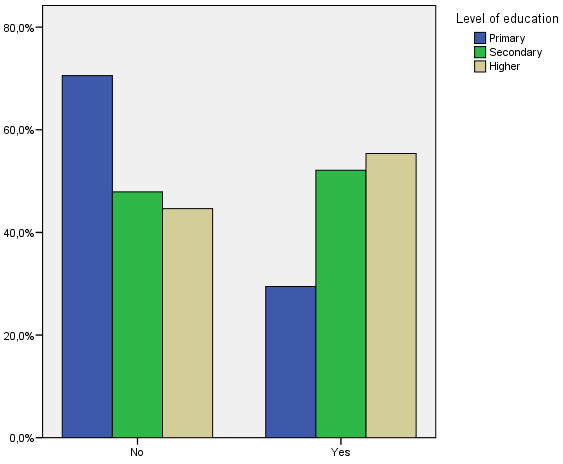
Relationship between the use of family planning methods and level of education.

The bivariate relationships shown in Table 4 indicate significant relationships at 95% of confidence regarding the use of family planning methods in the variables age, place of birth (see Figure 1), level of education, basic origin and having children under 14. The relationships indicate that those who use family planning methods less are older people (39 to 49 year-olds), Africans and Asians, people with primary school education (see Figure 2) and those without children. No statistically significant relationships are found with regard to sex, type of family planning methods used, income or perceptions of health.

**Figure 3:**
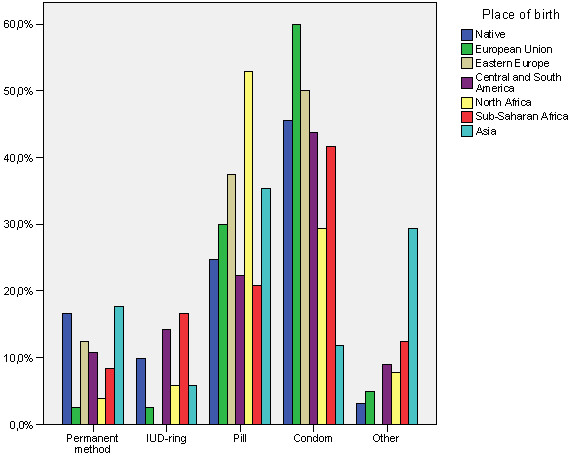
Relationship between the use of family planning methods used and place of birth.

Table 5 shows statistically significant relationships at 95% confidence with regard to type of family planning methods. No significant differences are found in the type of planning method used with regard to income or basic origin (natives and European Union versus economic immigrants) although differences do stand out when working with different origins of economic immigrants (see Figure 3). Thus, for example, it is worth highlighting that users from sub-Saharan Africa and Asian countries use traditional methods more frequently.

Finally, Table 6 shows the results of the statistical modelling. It is observed that individuals from North Africa, sub-Saharan Africa and Asia are less likely (36.68%, 38.59% and 70.51%, respectively) to use family planning methods than natives. That said, when we analyse this relationship taking into account users’ sex and origin, we observe that women from these geographical regions are more likely to use family planning methods than native men, although only Asian women show a statistically significant relationship in this respect (235% more likelihood than native men). Furthermore, users with secondary and university education have a greater likelihood of using family planning methods (30.59% and 39.29%, respectively) than individuals with primary education. People with children over 14 have a 35.35% more likelihood of using family planning methods than those without children. No significant relationships are detected with regard to perceived health status or chronic illness.

## Discussion

The article highlights the difference in use of family planning and contraception among different groups in a geographical region with a high rate of recent immigration where there is universal health cover and analyses the possible existence of sources of inequity in this different use of family planning methods.

Despite the fact that, as we have said, it is difficult to conclude whether the differences detected may be considered solely inequalities, in the sense that they reflect different patterns of behaviour and different preferences in the choice of family model due to sociological and cultural type reasons, we believe that the results do point, as Gillespie et al. [21] suggest, to the inequalities detected in this case being considered sources of inequity. Greater fertility rates among African and Asian populations, which according to the UNDP [37] comprise a proportion of economic immigrants to this country, along with less use of contraceptive methods (Table 7) suggest that the inequalities detected may be considered inequities in contraception in these populations when compared with the native population and populations from other geographical locations.

**Table 7:** Fertility rates by origin

**Users with children**	**%**	**mean**	**sd**	**min**	**max**
Natives	41.3	1.73	0.751	1	4
European Union	17.5	1.23	0.451	1	2
Eastern Europe	45.3	1.78	0.893	1	4
Central and South America	57,7	1.89	0.965	1	5
North Africa	61.3	2.11	1.064	1	6
Sub-Saharan Africa	67.2	2.42	1.579	1	8
Asia	56.3	2.13	1.390	1	5

The main limitations in the interpretation of these results relate to the use of a sample of public health services users during a specific period of time (year 2007) and not a random sample of the population and also are worth mentioning that the questionnaire used not provided detailed information regarding the individual incomes of these users. Despite said limitations, these results provide relevant information for planning improvements in the use of health services in this country.

## Conclusion

As follows from this analysis, greater or lesser use of family planning methods is related only to socioeconomic factors and not health status (real or perceived). Level of education, having children over 14 and geographical origin are elements that determine use. Even in Spain, a country with universal health service access, we have found sources of inequity. These results agree with the arguments proposed by Creanga [22], Davis [20], Igbal [19] and Gillespie [21], in the sense that although the use of family planning methods is first and foremost influenced by knowledge of and access to the same, gender roles, moral and/or cultural type reasons and individuals’ socioeconomic context also play an important role in this decision. This inequity is difficult to address from a solely medical point of view, as barriers to accessing family planning methods (difficulty with the language, dealing with health professionals, lack of information, etc.) refer to elements that must also be dealt with from a social viewpoint. Action in this respect would lead to a reduction in the number of undesired pregnancies and abortions, as well as fewer risks of infections and sexually transmitted diseases.

## Abbreviations

ABS, ÁreaBásica de Salud or Local Health Region; INLA, Integrated Nested Laplace Approximations; DIC, Deviance Information Criterion; CPO, conditional Predictive Ordinate; UNDP, United Nations Development Programme.

## Competing interests

The authors declare that they have no competing interests.

## Authors’ contributions

CS designed the fieldwork which provided the data and worked on data modelling and drafting the article, MS did the statistical modelling and worked on drafting and revising the article, LV participated in the fieldwork which provided the data, data cleansing and the descriptive analysis of the same, and also worked on drafting and revising the article. All the authors read and approved the final manuscript.

## GESIC collaborative group

Mercè Almirall^1,2^, Andrea Burón^3^, Xavier Cabré^1,2^, Dolors Corominas^4^, Pere Plaja^5^, Dolors Muñoz^6^, Marc Saez^7,8^, Carme Saurina^7,8^, Catalina Serna^9^, Laura Serra ^7,8^, Jorge Soler-González^9^, Esther Tobías^10^, Laura Vall-llosera^8^

^1^Biomedical Research Institut, Lleida (IRBLLEIDA), Spain

^2^University Hospital Universitari Arnau de Vilanova, Spain

^3^Hospital Mar-Parc de Salut MAR, Spain

^4^Institut d’AssistènciaSanitària de Girona (IAS), Spain

^5^Figueras Hospital, Spain

^6^University of Girona, Spain

^7^CIBER of Epidemiology and Public Health Spain

^8^ Research Group on Statistics, Econometrics and Health (GRECS), University of Girona, Spain

^9^ Regional Primary Care Management Office, Catalan Institute of Health, University of Lleida Spain

^10^ University Foundation of Bages (FUB), Spain
